# A SynBio explosion: a whole new world for Rubisco engineering

**DOI:** 10.1093/jxb/eraf189

**Published:** 2025-07-02

**Authors:** Dan Hong Loh, Laura H Gunn

**Affiliations:** Plant Biology Section, Cornell University, Ithaca, NY, USA; Plant Biology Section, Cornell University, Ithaca, NY, USA

**Keywords:** Carbon fixation, chaperones, chaperonins, photosynthesis, Rubisco, synthetic biology

## Abstract

This article comments on:

**Archer J, Kathpalia M, Lee B, Li S, Wang T.** 2025. Effects of chaperone selectivity on the assembly of plant Rubisco orthologs in *E. coli*. Journal of Experimental Botany **76**, 2809–2820 https://doi.org/10.1093/jxb/eraf140

This article comments on:

**Archer J, Kathpalia M, Lee B, Li S, Wang T.** 2025. Effects of chaperone selectivity on the assembly of plant Rubisco orthologs in *E. coli*. Journal of Experimental Botany **76**, 2809–2820 https://doi.org/10.1093/jxb/eraf140

**The first *Escherichia coli* synthetic biology (SynBio) system to successfully heterologously assemble chloroplast Rubisco represented the culmination of decades of Rubisco biogenesis research (** **Aigner *et al*., 2017** **). This system for Arabidopsis Rubisco ushered in a new era of higher throughput Rubisco studies, and has since been adapted for a limited number of other Rubiscos, accelerating both fundamental and applied Rubisco research. Archer *et al*. (2025) have developed new SynBio systems to assemble a larger variety of C**_**3**_ **Rubiscos and the first C**_**4**_ **and monocot Rubiscos, greatly expanding crop Rubisco engineering capabilities. Their explorations of chaperone selectivity and versatility could enable rapid production of SynBio systems for additional species.**

Rubisco is the gateway enzyme for CO_2_ fixation in photosynthetic organisms, often limiting photosynthesis and plant growth because of its slow catalytic turnover rate, poor substrate specificity for CO_2_, and ready inhibition (Carmo-Silva *et al*., 2015). To compensate for limited performance, leaves typically invest 30–50% of their soluble protein in Rubisco, which represents a significant nitrogen investment; leaves also open their stomata to obtain CO_2_, which comes with a water loss penalty. Thus, improving the catalytic performance of Rubisco could have profound implications for agricultural crop productivity and resource use efficiency.

A major limitation to Rubisco engineering is throughput. While Rubiscos of bacterial origin are relatively easy to assemble heterologously, Rubiscos from chloroplasts are completely reliant on the action of seven assembly factors (Fig. 1), which historically prevented rapid expression and engineering in microbial heterologous expression hosts, such as *Escherichia coli* or yeast. In contrast to microbial expression, modifying Rubisco in land plants is slow (Fig. 2). One approach to study and engineer Rubisco with higher throughput is to use Rubisco SynBio expression systems, which recapitulate the Rubisco biogenesis pathway in *E. coli*. Such systems have been used to identify the essential components of chloroplast Rubisco biogenesis (Aigner *et al*., 2017; Oh *et al*., 2024). They have also been used to demonstrate that differentially expressed Rubisco subunits confer different enzyme kinetics (Lin *et al*., 2020), that ancestrally reconstructed Rubiscos exhibit kinetic adaptation (Lin *et al*., 2022), and that altered Rubisco biogenesis in SynBio systems translates to altered Rubisco assembly in chloroplasts, albeit with certain caveats (Buck *et al*., 2023).

**Fig. 1.**
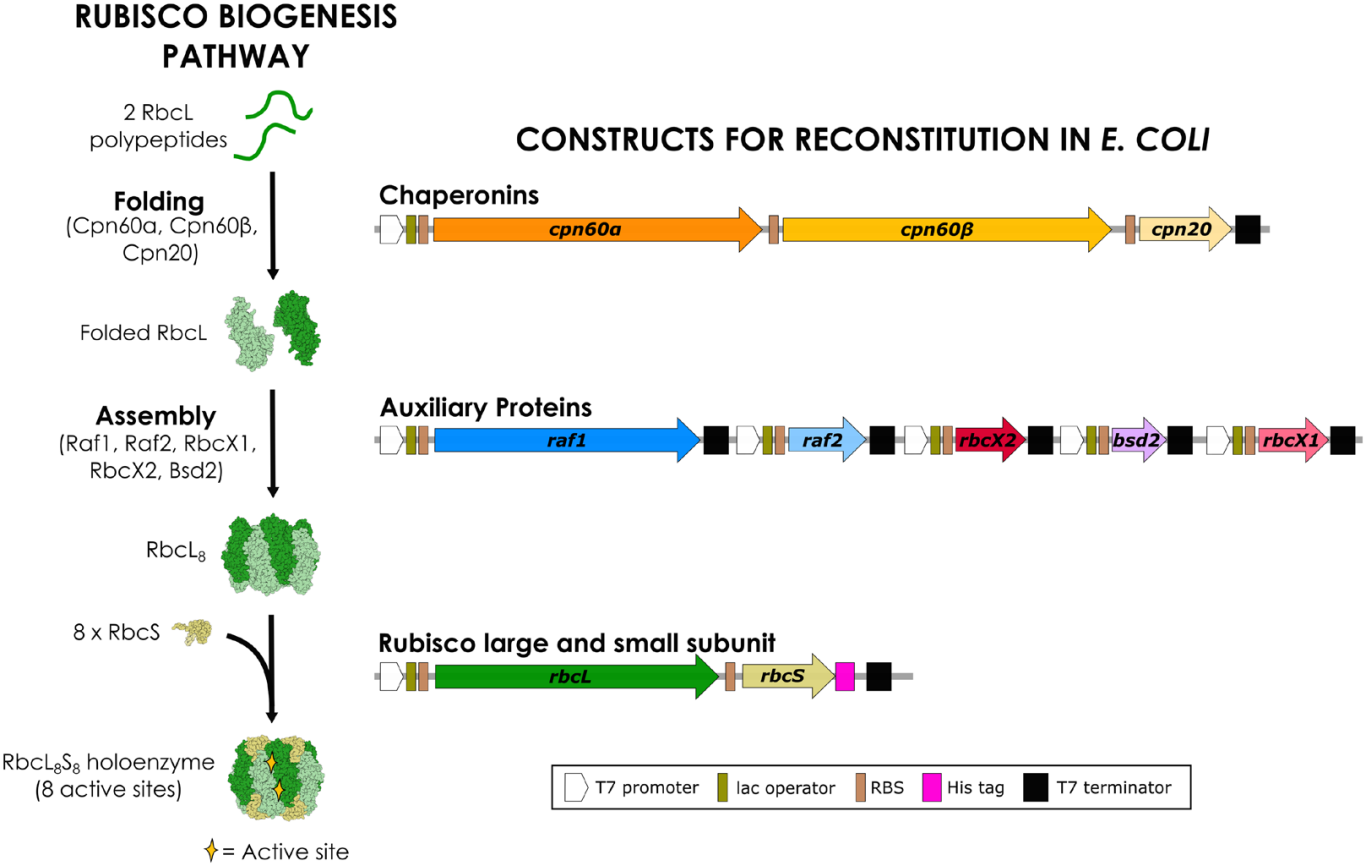
Rubisco SynBio expression systems reconstitute Rubisco biogenesis in *E. coli*. Several assembly factors (chaperonins and auxiliary proteins) are required during chloroplast Rubisco biogenesis to fold the Rubisco large subunit (RbcL), and assemble the Rubisco L_8_S_8_ holoenzyme (with the Rubisco small subunit; RbcS). Three chaperonin subunits (chaperonin 60ɑ, 60β, and 20) form an oligomeric cage that provides a favorable microenvironment to allow the chloroplast large subunit to fold. The auxiliary proteins [Rubisco accumulation factors 1 and 2 (Raf1, Raf2), RbcX1, RbcX2, and bundle sheath defective 2 (Bsd2)] stabilize L_2_ and L_8_ intermediate complexes. Finally, eight Rubisco small subunits assemble with the L_8_ complex to form the L_8_S_8_ Rubisco holoenzyme, which contains eight active sites. Co-expressing all the assembly factors with Rubisco-encoding genes in *E. coli* allows heterologous reconstitution of the Rubisco biogenesis pathway. Rubisco SynBio expression systems typically distribute the genes encoding Rubisco and assembly factors over 2–3 plasmids. Including a C-terminal affinity tag (e.g. a hexahistidine tag; His tag) on the Rubisco small subunit-encoding gene improves the ease of Rubisco purification and improves assembly capacity (Wilson *et al*., 2019). RBS: ribosome-binding site.

**Fig. 2.**
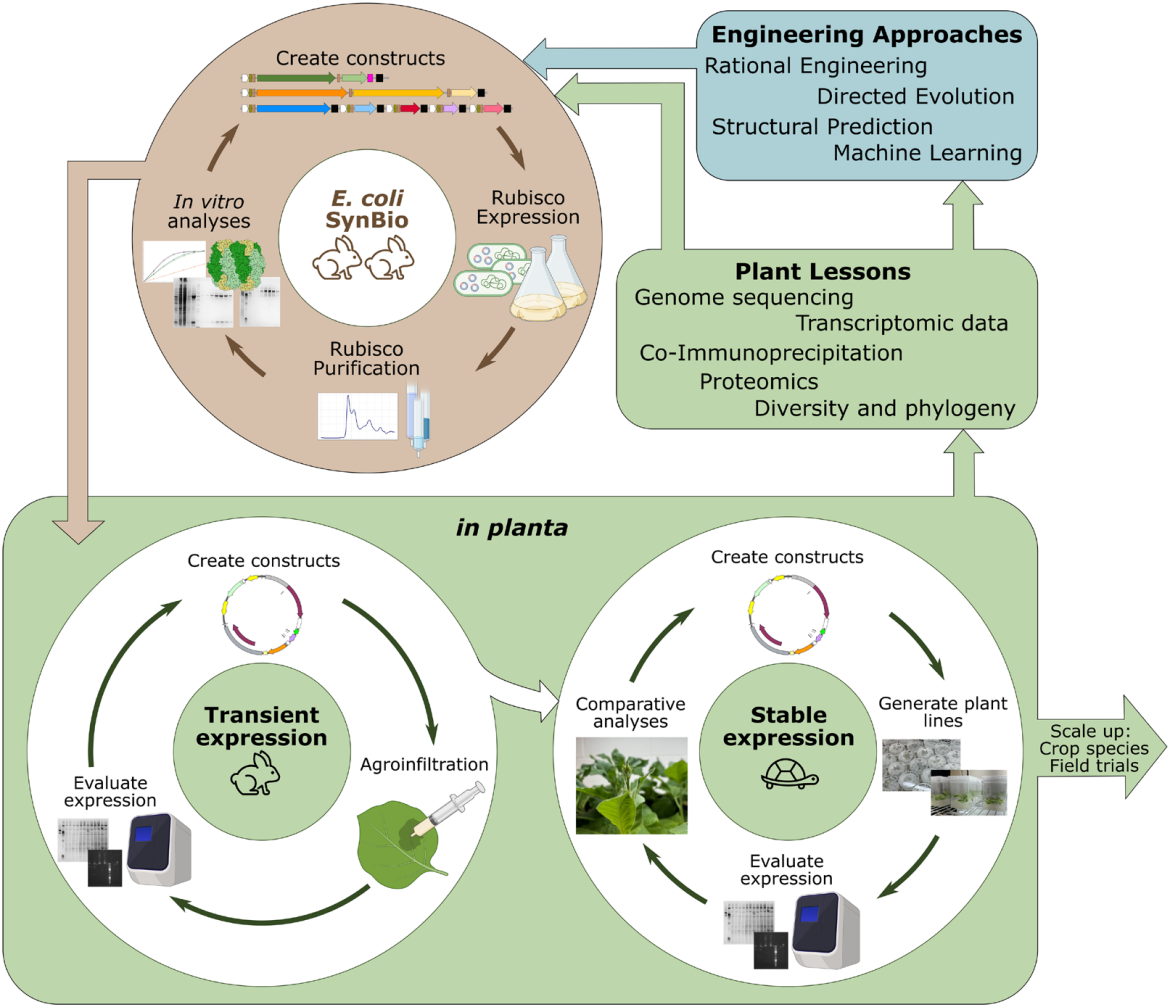
Accelerating fundamental and applied Rubisco research with an *E. coli* SynBio system. Rubisco SynBio *E. coli* expression systems are a significant part of a powerful toolbox for studying and engineering Rubisco. As shown in Fig. 1, Rubisco SynBio systems move the essential components of the Rubisco biogenesis pathway into *E. coli*. This allows this minimal system to be precisely modified with greater ease and throughput than *in planta* manipulation. Rubisco SynBio design is informed by ‘plant lessons’: genomic, transcriptomic, and proteomic data from plants that are used to obtain the correct Rubisco large and small subunit sequences, and identify the assembly factors required to assemble a given Rubisco. Information from plants can also be used to formulate hypotheses to test in SynBio systems. Engineering approaches can draw from a range of tools to decide how to modify Rubisco sequence–structure, which can then be screened by expressing and purifying it from *E. coli*, and its assembly capacity and kinetic performance can be evaluated with *in vitro* assays. Results from this initial screen, or ‘microbial lessons’, can be used to narrow down Rubisco candidates to be tested *in planta*, which has lower throughput, but increased biological relevance. For example, transient expression in leaves offers a medium-throughput first-pass validation of engineered Rubisco assembly and kinetic performance. Once the assembly and activity of modified Rubisco are verified via transient expression, stable chloroplast expression can be considered. This is the lowest throughput, but provides the most accurate data about how a modified Rubisco will perform in a chloroplast by allowing comparative analyses of plant growth, biomass, and leaf photosynthetic capacity. The performance of any promising plant lines needs to be tested in the field, before coordinating with plant breeders to incorporate and test promising Rubisco variants in elite crop varieties. Icons created in BioRender. Loh, D. (2025) https://BioRender.com/p75n012.

Despite the power and promise of Rubisco SynBio systems, these were available for a limited number of Rubiscos from C_3_ plants (Arabidopsis, tobacco, and certain hornwort Rubiscos). Archer *et al*. (2025) make significant contributions by expanding this capability for Rubiscos from multiple other C_3_ species (soybean, potato, cotton, and *Medicago truncatula*), as well as the first ever C_3_ and C_4_ monocot species (rice, barley, and maize) by using rice assembly factors. Monocot Rubiscos are of special interest because of the agronomic importance of key grain crops. However, Archer *et al*. (2025) only performed qualitative kinetic analyses to demonstrate that rice Rubisco is active in *E. coli*, while activities of the other assembled monocot Rubiscos remain to be tested.

Rubisco SynBio systems are only available for such a few species because chloroplast Rubiscos have co-evolved with their cognate assembly factors such that they generally cannot be assembled well with non-native assembly factors. While Lin *et al.* (2020) found that tobacco Rubisco could be assembled with certain combinations of Arabidopsis and tobacco assembly factors, the rules surrounding the versatility and/or selectivity of these factors are not well understood. A tobacco Rubisco SynBio system assembled hybrid Rubiscos comprising a foreign plant large subunit (closely related to tobacco) and the tobacco small subunit, where assembly capacity did not correlate with phylogenetic distance (Buck *et al*., 2023). Conversely, assembly capacity broadly correlated with phylogenetic distance when a SynBio system optimized for *Anthoceros agrestis* Rubisco was used to assemble other hornwort Rubiscos (Oh *et al*., 2024). Archer *et al*. (2025) demonstrate that Raf1 is one source of this selectivity. Various Rubiscos were able to be assembled with Arabidopsis assembly factors, as long as their cognate Raf1 was present. Chimeric Rubiscos containing a key species-specific Raf1 recognition sequence in Rubisco also enabled many (but not all) dicot C_3_ Rubiscos to be assembled by the Arabidopsis Rubisco SynBio system.

## SynBio-enabled fundamental discovery

Increased availability of Rubisco SynBio systems will allow higher throughput fundamental discovery. Since Rubisco SynBio systems pull the Rubisco biogenesis pathway out of the plant, and into *E. coli* (Fig. 1), they are a powerful tool for precise and high-throughput manipulation of Rubisco structure–function (by modifying the Rubisco-encoding genes) and biogenesis (by adding, removing, or modifying assembly factor sequences). This is especially significant because of the time it takes to perform experiments *in planta*, compared with *E. coli*, where plant transformation, especially when accessing the chloroplast genome, is the major bottleneck (Fig. 2).

Rubisco SynBio systems could allow us to crack the code for Rubisco assembly factor requirements. It is not known why some assembly factors are interchangeable or why the Raf1 recognition sequence is sometimes sufficient to permit folding of no-cognate Rubiscos (Archer *et al*., 2025). There is likely to be a complex, direct or indirect, interplay between assembly factors. For example, interactions with other chaperones upstream in the biogenesis pathway could influence the ability of a Rubisco to recognize a downstream chaperone. Indeed, chaperones have been observed to affect Rubisco structure, even after their displacement, in hornwort Rubisco (Oh *et al*., 2024). Alternatively, there could be additional recognition sequences required for Raf1 to recognize a given Rubisco. Future studies that systematically test these hypotheses could enable an understanding of the selectivity requirements for every assembly factor, allowing us to rapidly produce any Rubisco of interest in *E. coli*.

The power to specifically modify Rubisco-encoding genes with relatively high throughput and produce large quantities of pure protein also enables *in vitro* kinetic assays and biophysical characterization, including structural determination. Booms in cryoEM capabilities (Nakane *et al*., 2020) and structure prediction algorithms (Abramson *et al*., 2024) allow us to visualize and produce hypotheses about Rubisco structure–function with more power than ever before. More controlled dissection of these structure–function relationships in Rubisco, and broader catalytic surveys of understudied plant lineages, could deepen our understanding about the molecular evolution and constraints of the Rubisco catalytic mechanism (Young *et al*., 2016; Flamholz *et al*., 2019; Tcherkez and Farquhar, 2021; Bouvier and Kelly, 2023).

## SynBio-enabled engineering approaches for crop improvement

Rubisco SynBio systems provide a potential route to translational solutions for crop improvements. They allow Rubisco engineering on a scale never before imagined, and the timing of their availability coincides with other relevant technology expansions. Not only can Rubisco SynBio systems be used for higher throughput diversity screening of catalytically desirable Rubiscos, but they can also be used to screen engineered Rubisco variants, with only ‘winners’ being transferred to crops (Fig. 2). This approach allows us to bypass initial screening in a plant—where transformation capabilities are currently limited for most crop species.

Rubisco SynBio systems could be used for directed evolution of crop Rubiscos and/or their chaperones (Gionfriddo *et al*., 2024). Directed evolution requires generating a mutagenic library, and a method to select upon that library. Rubisco-dependent *E. coli* strains are already available (Zhou and Whitney, 2019), as are new capabilities to perform the mutagenic library step(s) *in vivo* (McDonald *et al*., 2025, Preprint). On the other hand, rational engineering of Rubisco has thus far been limited in its success. A contributing factor is the low throughput of plant transformation and thus relatively low data output to refine engineering approaches. Poorly understood long-range effects in Rubisco (Schulz *et al*., 2022) also makes predicting effects of amino acid changes on Rubisco performance seemingly impossible. While using artificial intelligence, especially machine learning, for rational design could currently be limited by insufficient independent Rubisco functional measurements, SynBio could offer a path to bridge that data gap. Indeed, machine learning-driven Rubisco engineering seems tantalizingly close, given preliminary success (Iqbal *et al*., 2023).

Translation of any enhanced Rubisco into crops has been made more precise with the advent, and improvements to the applicability and efficiency, of gene editing techniques, including the ability to now perform base editing of chloroplast-encoded genes (Nakazato *et al*., 2021). Fully realized translation of Rubisco improvements to crops for agricultural gains requires coordination with plant breeders to introduce improved Rubiscos into elite crop varieties (Fig. 2).

## Rubisco SynBio limitations

One limitation of Rubisco SynBio systems is the extent to which they can accurately mimic Rubisco assembly in a chloroplast environment. There is evidence of the tobacco system being a good proxy if chloroplast assembly is high (Buck *et al*., 2023), and current systems are clearly sufficient for Rubisco assembly. However, there could be additional factors that are involved in assembly that could tune Rubisco assembly or function but are not currently captured by the Rubisco SynBio systems, for example post-translational modifications (Valegård *et al*., 2018; Oh *et al*., 2023; Amaral *et al*., 2024). The ‘reliable proxy’ limitation could be mitigated by using complementary approaches, including intermediate screening for chloroplast Rubisco assembly and activity via transient expression, as well as the (much slower) stable integration into nuclear or chloroplast genomes of vascular plants (Fig. 2). Another challenge is the lack of a universal expression system for all Rubiscos-which is being actively addressed (e.g. Archer *et al*., 2025).

## Conclusion

SynBio Rubisco assembly has facilitated Rubisco research since its inception, quickly becoming an indispensable tool for Rubisco research allowing precise control of the Rubisco assembly conditions and permitting easy purification and high protein yields. Archer *et al*. (2025) build significantly on the existing tools by rapidly expanding the number of plant species amenable to Rubisco SynBio, and raise more questions and avenues for research to enable both fundamental discovery and translational opportunities. Rubisco SynBio, combined with other key technological breakthroughs including artificial intelligence, structure prediction, and chloroplast base editing, now provide real hope for designing and implementing Rubisco solutions in crops.

## Data Availability

No new data were generated or analysed in support of this research.
